# Ultrahigh Power Factor of Sputtered Nanocrystalline N‐Type Bi_2_Te_3_ Thin Film via Vacancy Defect Modulation and Ti Additives

**DOI:** 10.1002/advs.202403845

**Published:** 2024-08-09

**Authors:** Tingrui Gong, Lei Gao, Lingfeng Kang, Maolin Shi, Gu Hou, Shenghui Zhang, Dechao Meng, Juntao Li, Wei Su

**Affiliations:** ^1^ Microsystem & Terahertz Research Center China Academy of Engineering Physics Chengdu Sichuan 610200 China; ^2^ Institute of Electronic Engineering China Academy of Engineering Physics Mianyang Sichuan 621999 China

**Keywords:** annealing, Bi_2_Te_3_ thin film, magnetron sputtering, power factor, Ti additives

## Abstract

Magnetron‐sputtered thermoelectric thin films have the potential for reproducibility and scalability. However, lattice mismatch during sputtering can lead to increased defects in the epitaxial layer, which poses a significant challenge to improving their thermoelectric performance. In this work, nanocrystalline n‐type Bi_2_Te_3_ thin films with an average grain size of ≈110 nm are prepared using high‐temperature sputtering and post‐annealing. Herein, it is demonstrated that high‐temperature treatment exacerbates Te evaporation, creating Te vacancies and electron‐like effects. Annealing improves crystallinity, increases grain size, and reduces defects, which significantly increases carrier mobility. Furthermore, the pre‐deposited Ti additives are ionized at high temperatures and partially diffused into Bi_2_Te_3_, resulting in a Ti doping effect that increases the carrier concentration. Overall, the 1 µm thick n‐type Bi_2_Te_3_ thin film exhibits a room temperature resistivity as low as 3.56 × 10^−6^ Ω∙m. Notably, a 5 µm thick Bi_2_Te_3_ thin film achieves a record power factor of 6.66 mW mK^−2^ at room temperature, which is the highest value reported to date for n‐type Bi_2_Te_3_ thin films using magnetron sputtering. This work demonstrates the potential for large‐scale of high‐quality Bi_2_Te_3_‐based thin films and devices for room‐temperature TE applications.

## Graphical abstract

This work demonstrates the significance of incorporating Ti additives and post‐annealing to modulate defects and carrier transport in sputtered n‐type Bi_2_Te_3_ thin films. A 5 µm thick Bi_2_Te_3_ thin film achieves a record power factor of 6.66 mW mK^−2^ at room temperature. The inherent challenges of sputtered thin films are addressed and the thermoelectric properties of n‐type Bi_2_Te_3_ thin films are improved.

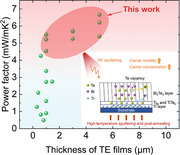

## 1 Introduction

The growth of wearable devices and microelectronics is driving the need for sustainable power supplies and on‐chip thermal management that can be integrated into electronic systems.^[^ ^1^ ^]^ Thin‐film thermoelectric devices (TFTEDs) are capable of achieving high power/cooling densities in compact packages and are promising for applications such as miniature power supplies, localized cooling, and high‐precision temperature control.^[^ ^2^ ^]^ The performance of TFTEDs is typically determined by the thermoelectric (TE) thin film, which is evaluated based on the dimensionless figure of merit ZT, defined as ZT = *α* ^2^ *T*/*ρk*. Where *α* is the Seebeck coefficient, *ρ* is the electrical resistivity, *k* is the total thermal conductivity, and *T* is the absolute temperature. To fully exploit the power generation or cooling potential of a TFTED, a sufficiently large power factor (*α* ^2^/*ρ*) and a sufficiently small *k* are required to maximize the ZT.^[^ ^3^ ^]^ The average ZT of a TE thin film determines the energy conversion efficiency of a TFTED, while the power factor (PF) directly determines the output power density and cooling density at a given operating boundary condition.^[^ ^3^, ^4^ ^]^ Output power and cooling power are the key metrics used to advertise the performance of TE devices in the commercial market. As such, PF is commonly used to evaluate TE performance instead of ZT.

Generally, optimization of the alloy composition and microstructure of TE materials can improve their TE performance. Low‐dimensional controllable growth of thin film crystals can be achieved by modulating the carrier concentration and electronic energy band structure. Effective methods for achieving this include modifying the energy level structure,^[^ ^5^ ^]^ heavy doping,^[^ ^6^ ^]^ quantum potential wells,^[^ ^7^ ^]^ and electron energy barrier filtering.^[^ ^8^ ^]^ Improving TE performance also involves improving conventional materials (changing material composition),^[^ ^9^ ^]^ researching and testing new materials (metal oxide materials, polymeric materials),^[^ ^10^ ^]^ conducting process research (fabrication process improvements, superlattice materials, surface treatments),^[^ ^11^ ^]^ and conducting computational research based on first principles.^[^ ^12^ ^]^ Over the past two decades, there have been significant improvements in ZT, mainly due to the discovery of new nanostructured TE materials and the implementation of innovative strategies.^[^ ^13^ ^]^ However, these optimization strategies have limitations in improving the PF of TE materials.

Bi_2_Te_3_‐based alloys are currently the most effective TE materials at room temperature and have a wide range of commercial applications. However, there are still challenges that need to be addressed, such as miniaturization of bulk materials and preparation of high‐quality TE thin films. Common methods for preparing TE thin films include molecular beam epitaxy (MBE), metal organic chemical vapor deposition (MOCVD), physical vapor deposition (PVD), and electrochemical deposition (ECD). However, most of these techniques for preparing TE films often suffer from low deposition rates and high costs, which are not conducive to commercialization. Among these methods, low‐cost ECD can be used to prepare thick TE films. However, the literature indicates that TE films prepared by ECD method have fluffy morphology, poor crystallinity and low density, resulting in poor TE performance.^[^ ^14^ ^]^ PVD‐based TE thin films offer several advantages, including easy composition control, high quality, easy thickness control, good reproducibility and scalable production.^[^ ^15^ ^]^ Optimization of substrate material,^[^ ^16^ ^]^ sputtering pressure,^[^ ^17^ ^]^ substrate temperature,^[^ ^18^ ^]^ material modification,^[^ ^19^ ^]^ film thickness,^[^ ^20^ ^]^ and post‐annealing conditions can significantly improve the PF of TE thin films.^[^ ^21^ ^]^ Other methods for maximizing the PF include optimizing the carrier concentration through doping with Cu, Mn, Ag, and other dopants, thus breaking the equilibrium between *S* and *σ*.^[^ ^22^ ^]^ It is worth noting that magnetron sputtering has significant advantages over traditional physical fabrication techniques such as screen printing, ink printing, co‐evaporation, vacuum filtration, etc. Magnetron sputtering is capable of fabricating homogeneous TE films with excellent performance on various substrates, precisely controlling the thickness and achieving good adhesion, which can result in TE films with high power factor and surface smoothness.^[^ ^15^, ^23^ ^]^ For instance, Cu_2_Se thin films fabricated by magnetron sputtering have been demonstrated to exhibit an exceptional power factor of 6 mW mK^−2^.^[^ ^24^ ^]^ However, the thermal co‐evaporation technique presents certain challenges in terms of substrate selection and component control. Typically, the PF of n‐type Bi_2_Te_3_ thin films prepared using the thermal co‐evaporation technique can reach 4.87 mW mK^−2^, which is one of the best results reported in the literature.^[^ ^18b^ ^]^ Consequently, magnetron sputtering is a suitable method for fabricating TE thin films with a dense and stable structure and satisfactory TE properties.

Nevertheless, thin films prepared by magnetron sputtering tend to have a lower PF than those prepared by thermal evaporation techniques.^[^ ^25^ ^]^ Lattice mismatch is a common problem in sputtered films, especially as film thickness increases. This can lead to reduced bond strength, high internal stresses, and more defects in the epitaxial layer, ultimately leading to degraded crystal quality and film chipping.^[^ ^26^ ^]^ Therefore, sputtered films cannot be too thick. However, very thin TE films are not ideal for creating a temperature difference between the two ends, which can result in poor device performance.^[^ ^27^ ^]^ Consequently, improving the TE performance of sputtered films, especially the PF, is extremely difficult. Strategies to improve the PF of n‐type Bi_2_Te_3_ thin films still face serious challenges such as low performance, poor stability, and process complexity. In order to facilitate sustainable energy harvesting and on‐chip cooling, it is essential to investigate cost‐effective and reproducible methods for fabricating thin films with high PFs. To mitigate the lattice mismatch of Bi_2_Te_3_ thin films, it is often necessary to deposit an adhesion layer at the interface to improve adhesion. Commonly used adhesion layer materials include Ti, Cr, and TiW, among which the CTE of Ti (≈8.6 × 10^−6^ K^−1^) is closest to that of BiTe (≈14‐18 × 10^−6^ K^−1^). Meanwhile, Ti has a low Young's modulus and is capable of large elastic deformation under low stress, which can partially compensate for the difference in expansion or contraction caused by the CTE mismatch, thus relieving the interfacial stress and maintaining the structural stability.^[^ ^28^ ^]^ Ti has also been demonstrated to be able to induce low contact resistance and excellent mechanical properties at the same time, making it an ideal candidate for realizing low contact resistance in Bi_2_Te_3_‐based TFTEDs.^[^ ^29^ ^]^

**Figure 1:**
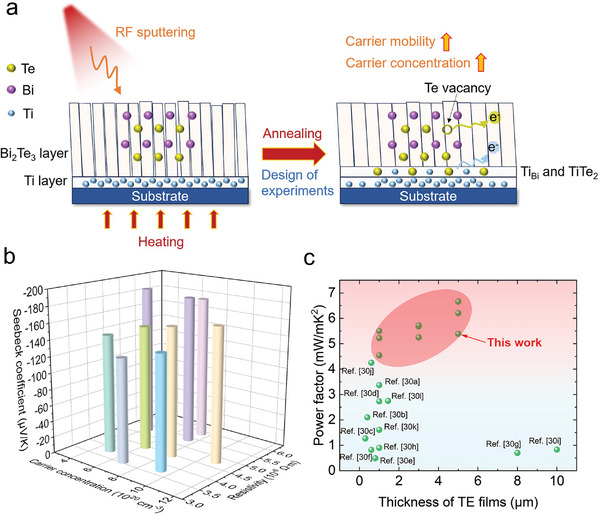
a) Schematic of the mechanism for optimizing the thermoelectric performance of sputtered n‐type Bi_2_Te_3_ thin films. b) Carrier concentration, resistivity, and Seebeck coefficient of n‐type Bi_2_Te_3_ thin films at room temperature. c) Comparison of power factor of sputtered n‐type Bi_2_Te_3_ thin films in this work and other representative works.^[^ ^30^ ^]^

Here, we present nanocrystalline n‐type Bi_2_Te_3_ thin films with an average grain size of ≈110 nm prepared using magnetron sputtering and post‐annealing. High‐temperature deposition and annealing processes can exacerbate Te evaporation and generate Te vacancies, which can create electron‐like effects and increase the carrier concentration. Carrier mobility is reduced due to the increased chance of lattice collisions, resulting in a lower velocity per unit number of electrons. Raising the annealing temperature leads to larger grain size, which also causes the fusion of grain boundaries, internal interfaces, and film defects, resulting in denser films with higher mobility. In addition, the high‐temperature treatment activates the ionization of pre‐deposited Ti additives at the bottom of the film. These Ti ions partially diffuse into Bi_2_Te_3_, causing substitution defects between Ti and Bi, resulting in an electron‐effect that further optimizes carrier concentration. This also allows the epitaxial growth of Bi_2_Te_3_ on the “transition layer” of TiTe_2_ to form highly (015) oriented films with improved electrical properties (**Figure** **1**a). By modulating the vacancy defects and introducing Ti additives, it is possible to simultaneously increase both the carrier concentration and the carrier mobility of the thin films. The process parameters, including film thickness, annealing temperature, and annealing time, are optimized using the Design of Experiment (DoE) method. The sputtered n‐type Bi_2_Te_3_ thin films exhibit a maximum carrier concentration of 1.09 × 10^21^ cm^−3^, a maximum mobility of 33.3 cm^2^/V∙s, and a minimum resistivity of 3.56 × 10^−6^ Ω∙m as well as a maximum absolute Seebeck coefficient of 187.3 µV K^−1^ at room temperature (Figure 1b). Consequently, the PFs of the sputtered Bi_2_Te_3_ thin films in this work are higher than those reported for state‐of‐the‐art Bi_2_Te_3_ thin films. Notably, a 5 µm thick n‐type Bi_2_Te_3_ thin film can achieve a record PF of 6.66 mW mK^−2^ (Figure 1c).^[^ ^30^ ^]^ This work highlights the significance of incorporating Ti additives and post‐annealing to modulate defects and charge carrier transport in sputtered n‐type Bi_2_Te_3_ thin films, which can facilitate their scaled‐up application for sustainable energy harvesting and on‐chip cooling.

## 2 Results and Discussion

**Figure 2:**
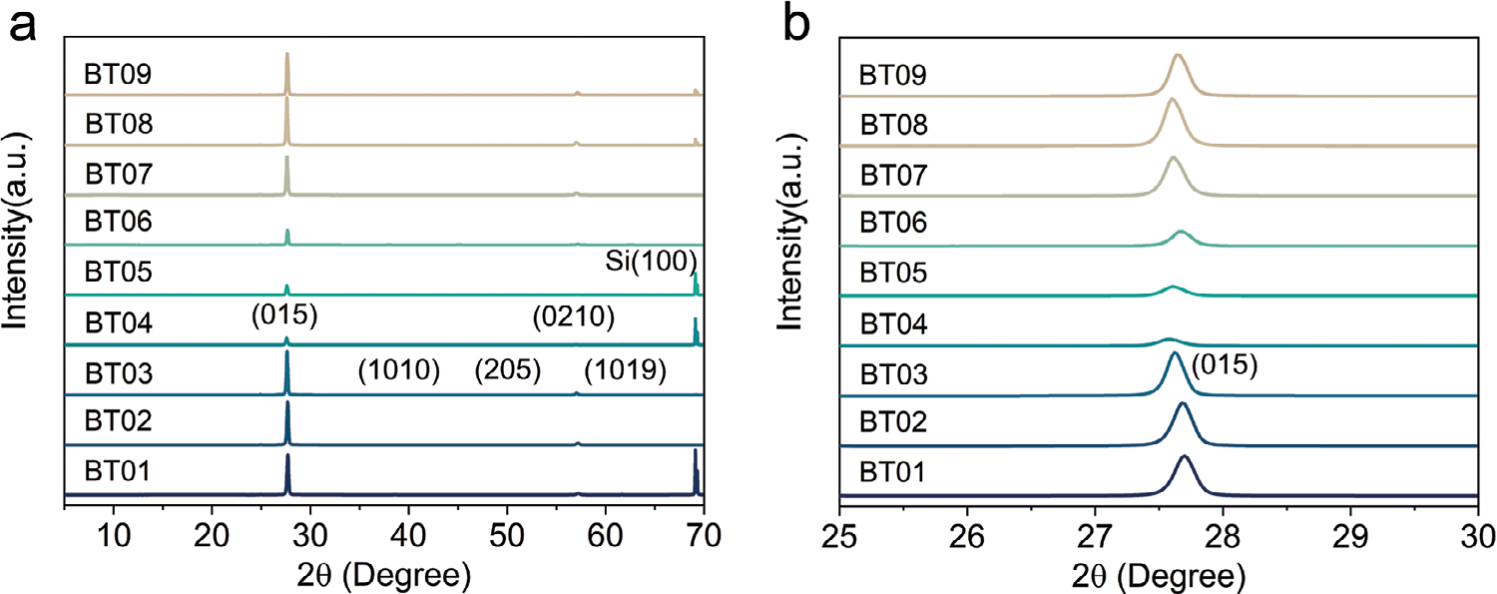
The XRD patterns of n‐type Bi_2_Te_3_ thin films. a) The nine samples. b) Enlarged image from 25° to 30°.

The process control parameters such as film thickness, annealing temperature and annealing time for sputtered n‐type Bi_2_Te_3_ thin films are investigated using the DoE method (Table S1, Supporting Information). Nine experimental schemes are designed using a three‐factor, three‐level orthogonal array (Table S2, Supporting Information) to investigate the electrical and TE properties of the Bi_2_Te_3_ thin films. The samples are labeled according to the experimental schemes. **Figure** **2** presents the XRD patterns of the nine Bi_2_Te_3_ thin films. The patterns exhibit no additional Bragg reflection peaks due to impurities, and all peaks correspond to Bi_2_Te_3_ and Si (Figure 2a). The dominant grains in all nine samples are those with (015) planes, as indicated by the main peak of (015) in the XRD patterns. This is in agreement with previous literature.^[^ ^16^, ^18^, ^20^, ^29^, ^31^ ^]^ In addition, the intensity of the (015) peak is stronger for film thicknesses of 1 and 5 µm. Weak peaks such as (0210), (101), (1010), (205), and (1019) can be indexed to the standard diffraction pattern of Bi_2_Te_3_ (JCPDS No. 15–0863). The (015) peak of the 1 µm thick films (BT01, BT02, and BT03) shifts slightly to the left with increasing annealing temperature and time (Figure 2b), indicating an increase in the lattice constant according to Bragg's law. Annealing may have promoted the formation of larger grains, significantly increasing the crystallinity and thus changing the lattice constant. However, increasing the annealing temperature results in a slight rightward shift of the (015) peaks for both the 3 and 5 µm samples. This shift indicates a decrease in the lattice constant, which may be caused by the difference in annealing time.

**Figure 3:**
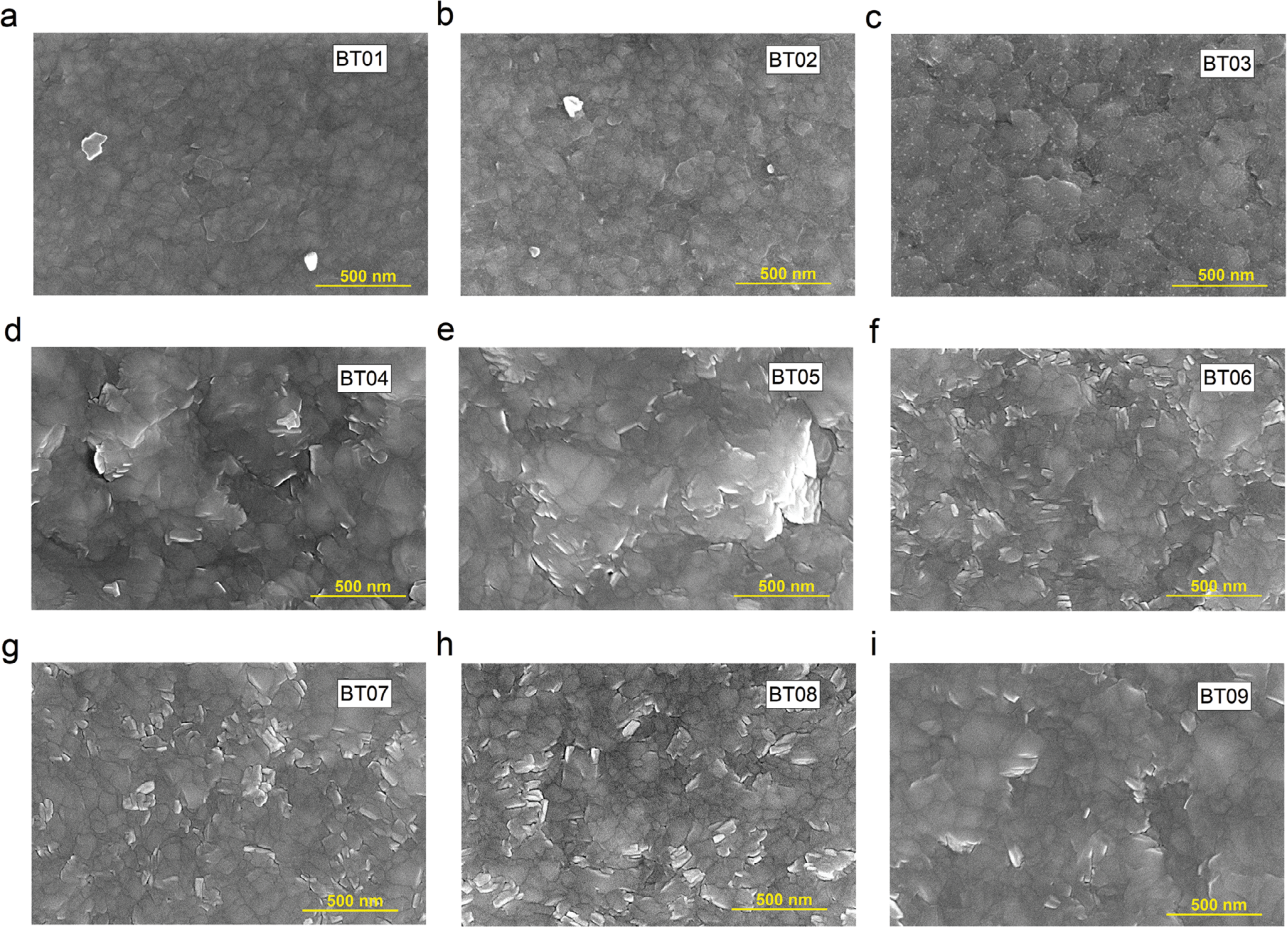
Surface SEM images of the nine Bi_2_Te_3_ thin films, showing highly dense nanograined structures with a minimum size of <100 nm. a) BT01. b) BT02. c) BT03. d) BT04. e) BT05. f) BT06. g) BT07. h) BT08. i) BT09.

The surface morphology and microstructural evolution of the nine samples are depicted in **Figure** **3**. It is evident that nanograined Bi_2_Te_3_ thin films are naturally formed after high‐temperature deposition (270 °C) and post‐annealing. No amorphous intercalation is observed and the minimum grain size is less than 100 nm. The grains are highly crystalline, but with different lattice orientations, indicating that the thin films are polycrystalline. As the film thickness increases from 1 to 5 µm, the surface becomes less flat and the grain boundaries become more defined while some holes and defects may form. However, annealing can increase the grain size, resulting in a more uniform distribution and suppressed fluctuation of the barrier height. This is beneficial for improving carrier mobility and Seebeck coefficient.^[^ ^32^ ^]^ Figure S1 (Supporting Information) provides additional surface SEM images of the nine samples at a 5 µm scale. The thin films with a thickness of 1 µm exhibit the highest density. Besides, all 3 µm thin films show distinct grain boundaries. As the film thickness increases to 5 µm, the grain size becomes more uniform, resulting in a highly dense film. The average grain size distributions of the nine Bi_2_Te_3_ thin films are compared in Figures S2 and S3 (Supporting Information), which are calculated from Figure S1 (Supporting Information). The average grain size of all samples is ≈110 nm. Specifically, BT01 has the smallest average grain size of 86.88 nm. Furthermore, the average grain size increases significantly with higher annealing temperatures.

The atomic percentages of Bi and Te in the Bi_2_Te_3_ thin films, as determined by surface EDS analysis, are listed in Table S3 (Supporting Information). The percentages do not match those of the Bi_2_Te_3_ source target (Bi:Te = 2:3). Ti is not detected in the EDS analysis, most likely due to the thinness (50 nm) of the Ti adhesion layer.^[^ ^33^ ^]^ Both thin films show Te vacancies resulting from Te evaporation during high‐temperature sputtering and annealing.^[^ ^34^ ^]^ The evaporation energy of Te (52.55 kJ mol^−1^) is much lower than that of Bi (104.80 kJ mol^−1^), making Te easier to evaporate than Bi.^[^ ^22a^ ^]^ It is important to note that the Te contents of BT02 and BT03 are only 53.7% and 50.1%, respectively. The most significant evaporation of Te occurs in BT03, which is subjected to the highest annealing temperature (350 °C) and the longest annealing time (4 h). Figure S4 (Supporting Information) illustrates the mean main effect of the control parameters on the composition of the thin film. As the film thickness increases, the atomic percentage of Bi decreases while the atomic percentage of Te increases. Although increasing the film thickness can partially restore the Te content, the amount of restoration still depends on the annealing conditions. Furthermore, with an increase in annealing temperature and time, the atomic percentage of Bi increases while the atomic percentage of Te decreases. This confirms that the high‐temperature annealing process exacerbates the evaporation of Te, resulting in the lowest Te content in BT03.

**Figure 4:**
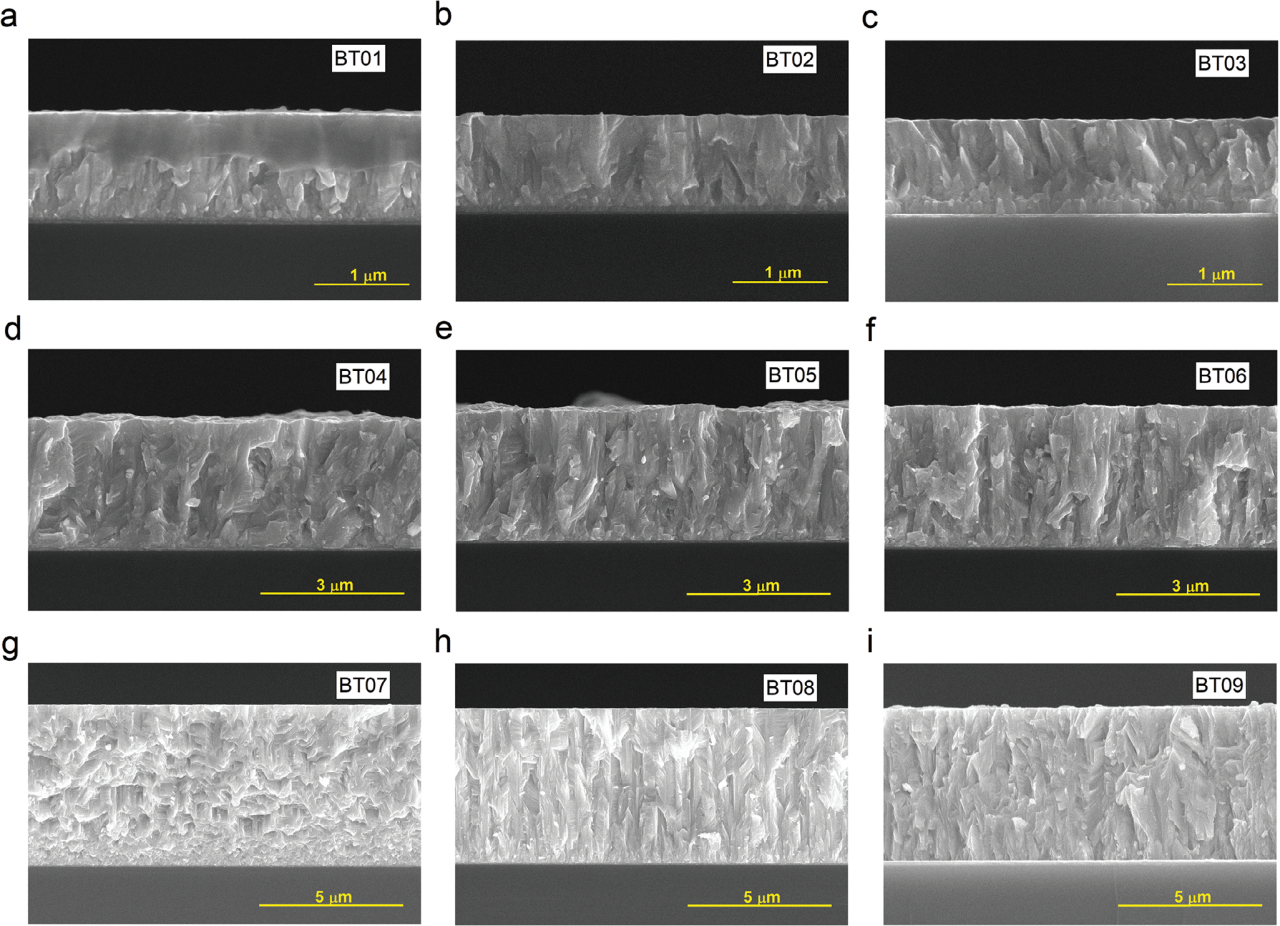
Cross‐sectional SEM images of the nine Bi_2_Te_3_ thin films, showing dense columnar single crystal structures with high (015) selective orientation. a) BT01. b) BT02. c) BT03. d) BT04. e) BT05. f) BT06. g) BT07. h) BT08. i) BT09.

The cross‐sectional SEM images of the nine samples are shown in **Figure** **4**. All thin films exhibit a dense columnar structure with high (015) selective orientation. The roughness of the films increases significantly with film thickness. However, the annealing treatment leads to further recrystallization of the films, resulting in a dense, large columnar single crystal structure. The diffusion of the Ti layer into Bi_2_Te_3_ near the substrate in the 1 µm film becomes more pronounced with increasing annealing temperature and time, as shown in BT03. To investigate whether Ti diffuses into Bi_2_Te_3_, the time‐of‐flight secondary ion mass spectrometry (Tof‐SIMS) mapping of BT03 is further depicted in Figures S5 and S6 (Supporting Information). The ion excitation depth exceeds the thickness of BT03, reaching 1.5 µm. The intensity of Ti ions increases rapidly at a depth of ≈500 nm. At this location, the intensity of Te ions increases slightly, while the intensity of Bi ions decreases drastically. This suggests that Ti atoms may have partially or completely replaced the Bi sites to form a transition layer.

**Figure 5:**
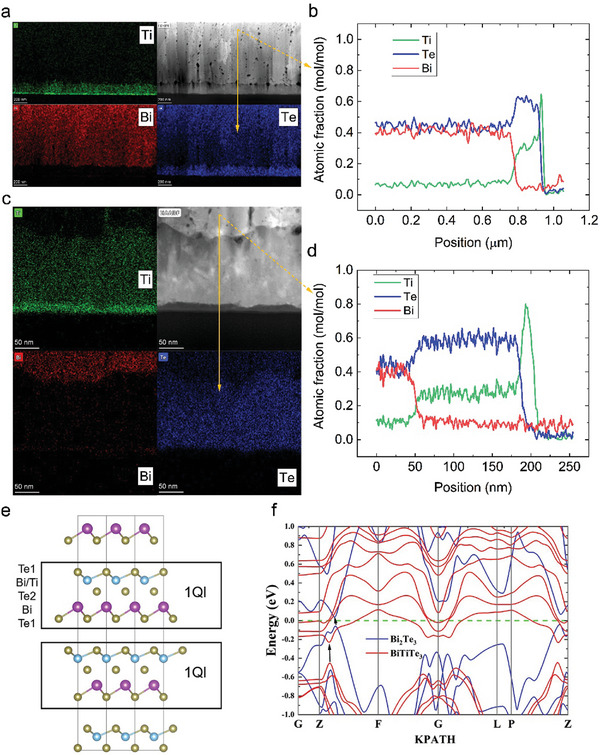
Cross‐sectional HAADF‐STEM images of the BT03 sample and calculated band structure of Ti‐doped Bi_2_Te_3_. a) Low magnification bright field image of STEM HAADF and corresponding EDS elemental mapping. b) Corresponding compositional profile along the arrow line. c) High‐magnification bright‐field image of STEM HAADF and EDS elemental mapping, showing a clear diffusion of Ti by nearly 150 nm and that the diffusion region is rich in Te but almost devoid of Bi. d) Compositional profile along the arrow line, showing that the atomic ratio of Te to Ti is close to 2:1. e) Lattice structure of Ti‐doped Bi_2_Te_3_. f) Band structure of Ti‐doped Bi_2_Te_3_.

The diffusion behavior of Ti in the Bi_2_Te_3_ thin film is further revealed by nanoscale investigations using cross‐sectional TEM. **Figure** **5** displays the high‐angle annular dark‐field scanning transmission electron microscopy (HAADF‐STEM) image of the BT03 sample along with the corresponding high and low magnification EDS images. The images demonstrate the upward diffusion of Ti near the Si substrate. The Ti diffusion region still contains a Te‐rich layer with almost no Bi present. The cross‐sectional HAADF‐STEM image of BT09 (Figure S7, Supporting Information) also confirms that Ti atoms may have replaced the Bi sites, creating substitution defects and possibly acting as electron donors. The compositional profile in Figure 5 also quantitatively confirms that Ti diffuses upward by almost 150 nm. Moreover, the energy band structure of Ti‐doped Bi₂Te₃ was calculated, which revealed that the lattice constant and bandgap of the system are changed. The values of these parameters are given in Table S4 (Supporting Information). The replacement of Bi sites by Ti atoms results in an upward shift of the Fermi energy level (green line) into the conduction band, which confers metallicity and electron donation to the system, leading to a significant increase in the electrical conductivity. Furthermore, at the Z‐F symmetry point, the conduction band minimum (CBM) increases while the valence band maximum (VBM) decreases, resulting in an increase in the bandgap (*E*_g_). The optimum thermoelectric performance at the optimum operating temperature is limited by the bandgap due to intrinsic excitation. According to the Goldsmid‐Sharp relationship, *E*_g_ = 2*eS*_max_ *T*(*S*_max_),^[^ ^35^ ^]^ an increase in Eg results in an increase in the peak Seebeck coefficient. The bandgap at gamma is smaller compared to the undoped energy bands, indicating a tendency for multi‐level degeneracy in the energy bands, which can further increase the Seebeck coefficient. This conclusion is consistent with previous findings in the literature.^[^ ^36^ ^]^ Meanwhile, it is clear that the concentration of Bi in the Ti diffusion region is negligible, and the atomic ratio of Te to Ti is ≈2:1, suggesting the possible formation of a TiTe_2_ transition layer. It should be noted that TiTe_2_ is not detected in Figure 2, mainly because the TiTe_2_ transition layer is located at the bottom of the Bi_2_Te_3_ thin film, which may have a lower intensity. The formation of TiTe_2_ can be further indicated by taking the logarithm of the vertical coordinates of the XRD patterns of samples BT03 and BT09 (Figure S8, Supporting Information). TiTe_2_ is known as a prototype of a Fermi liquid due to its behavior as a low resistivity semimetal.^[^ ^37^ ^]^ Sun et al. have recently reported that due to the rapid diffusion rate of the reaction between Te and Ti, a broad region of Te‐ and Ti‐rich material was created in close proximity to the BiTe interface, resulting in the formation of the TiTe_2_ reaction layer. Given the negligible potential barrier between the semimetallic TiTe_2_ and metallic Ti, and the fact that the heavily doped BiTe reduces the barrier width, it is possible for the Ti/BiTe interface to form an ohmic contact with low contact resistivity.^[^ ^28^ ^]^ More importantly, the TiTe_2_ reaction layer can facilitate metallurgical bonding and reduce internal stress, thereby ensuring high bonding strength of the interfacial structure. Thus, the epitaxial growth of Bi_2_Te_3_ on the “transition layer” of TiTe_2_ is ensured to form highly (015) oriented films with improved electrical properties. Figure S9 (Supporting Information) further shows the high‐resolution TEM (HRTEM) images of samples BT03 and BT09. High‐temperature annealing can cause the film grain boundaries to fuse, making it difficult to detect internal interfaces and defects. This process can reduce carrier concentration while increasing mobility. The Bi_2_Te_3_ crystals are composed of five layers of building blocks, each containing five atomic planes in the order Te1‐Bi‐Te2‐Bi‐Te1. The building blocks are held together by weak van der Waals forces. The Bi_2_Te_3_ grains exhibit a strong (015) texture, which is consistent with the XRD test results.

**Figure 6:**
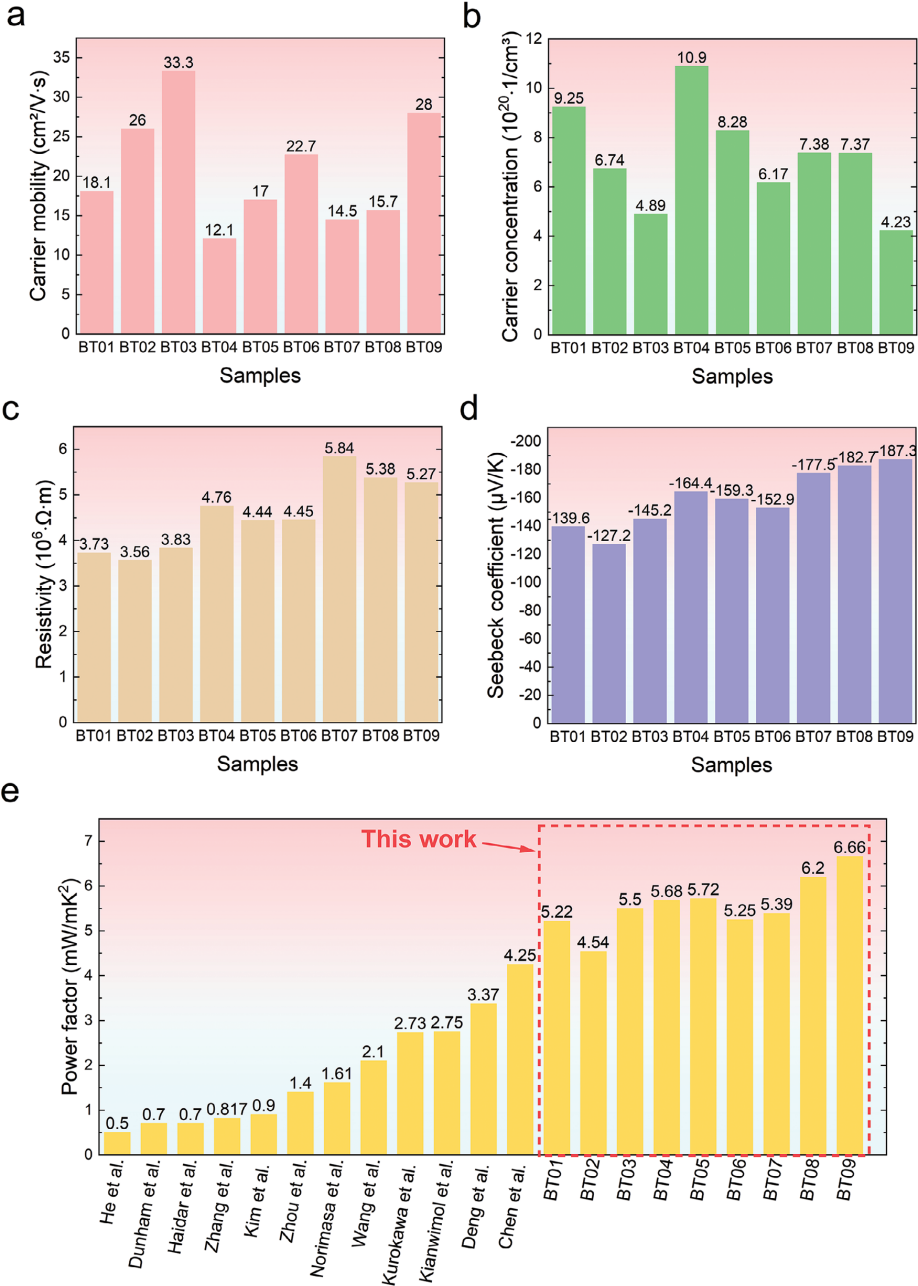
Electrical and TE properties of the nine Bi_2_Te_3_ thin films at room temperature. a) Carrier mobility. b) Carrier concentration. c) Resistivity. d) Seebeck coefficient. e) Comparisons of the power factors achieved in this work with those of the previous reports on sputtered n‐type Bi_2_Te_3_‐based thin films.^[^ ^30^ ^]^

**Figure** **6** illustrates the electrical and TE properties of Bi_2_Te_3_ thin films at room temperature for nine different schemes. The mean of signal‐to‐noise (S/N) ratios of the carrier mobility and the factor effect plots (Figure S10a,b, Supporting Information) demonstrate that the annealing temperature has the largest effect on the carrier mobility, followed by the film thickness and the annealing time. Specifically, increasing the annealing temperature results in higher carrier mobility (Figure 6a). Raising the annealing temperature can enlarge the grain size in Bi_2_Te_3_, resulting in a more uniform grain size distribution. This can also reduce the number of charged defects and result in denser films due to the fusion of grain boundaries, internal interfaces, and defects in the film. Among the tested samples, BT03 exhibits the highest carrier mobility of 33.3 cm^2^/V∙s, which can be attributed to its higher annealing temperature and longer annealing time. The carrier concentration of BT03 is only 4.89 × 10^20^ cm^−3^. In contrast, samples with lower annealing temperatures have higher carrier concentrations, probably because the finer nanograins increase the concentration of charged defects.^[^ ^38^ ^]^ Carrier mobility is reduced due to the increased chance of lattice collisions, resulting in a lower velocity per unit number of electrons. The observed trend between carrier mobility and carrier concentration is consistent with the existing literature and within the expected limits.^[^ ^39^ ^]^ Similar to mobility, carrier concentration is significantly affected by annealing temperature (Figure S10c,d, Supporting Information). As the annealing temperature increases, the carrier concentration decreases (Figure 6b). The lowest carrier concentration of 4.23 × 10^20^ cm^−3^ is found in BT09. Nevertheless, all nine samples have carrier concentrations within the optimal range of 10^19^–10^21^ cm^−3^.^[^ ^40^ ^]^ This is due in part to the evaporation of Te caused by the high‐temperature sputtering and annealing processes, which provide sufficient carrier concentration. The evaporation of each Te results in a Te vacancy with two free electrons, as shown in the following equation^[^ ^22a^ ^]^

(1) $$Bi_{2}Te_{3}=2Bi_{Bi}^{\times }+\left(3-x\right)Te_{Te}^{\times }+xTe\left(g\right)↑+xV_{Te}^{2+}+2xe^{-}$$

Te vacancies are electron‐like defect states that can increase the carrier concentration and act as n‐type dopants. For polycrystalline samples with nanocrystalline grains, the dangling bonds at the grain boundaries due to Te defects can also be considered as part of the Te vacancy concentration and contribute to n‐type doping.^[^ ^22a^ ^]^ Furthermore, the Ti additives also play an important role in increasing the carrier concentration. We also prepared 1 µm thick Bi_2_Te_3_ thin films with and without Ti additives and compared their electrical transport properties (Figure S11, Supporting Information). The results confirm that Ti acts as an electron donor, increasing the carrier concentration, but also slightly decreasing the carrier mobility. In conjunction with annealing, carrier mobility and carrier concentration can be increased simultaneously, resulting in a significant reduction in resistivity. Electrical conductivity is directly proportional to the product of carrier mobility and carrier concentration (*σ* = *neµ*), where *µ* is the carrier mobility, *e* is the elementary charge, and *n* is the carrier concentration. Sample BT02 has the highest product of carrier mobility and carrier concentration, resulting in the lowest resistivity of 3.56 × 10^−4^ Ω∙cm (Figure 6c). It is important to note that the resistivity of the thin film is significantly affected by its thickness, as shown in Figure S10e,f (Supporting Information). This is due to the fact that films of different thicknesses have different crystal densities and surface roughness (Figure 3). Therefore, the resistivity range is affected by the thickness of the film, which is determined by factors such as grain size, porosity, and defects. Furthermore, the Seebeck coefficient is mainly influenced by the film thickness (Figure S10g,h, Supporting Information). The samples with a film thickness of 5 µm have higher Seebeck coefficients than the others (Figure 6d). BT09 has the highest absolute value of Seebeck coefficient at 187.3 µV K^−1^. This can be attributed to the fact that the increase in film thickness favors the formation of a temperature difference between the two ends of the film, which can positively affect the Seebeck potential. The Seebeck coefficient is inversely proportional to the carrier concentration and can be expressed by the following equation^[^ ^40b^ ^]^

(2) $$S=\frac{8\pi^{2}k_{B}^{2}}{3eh^{2}}{\left(\frac{\pi }{3n_{c}}\right)}^{2/3}m_{d}^{*}T$$

where *k*_B_ is the Boltzmann constant, *h* is the Planck constant and $m_{d}^{*}$ is the DOS effective mass. Samples with lower carrier concentrations generally have better Seebeck coefficients, but this is not always the case. The Seebeck coefficient is influenced not only by the carrier concentration but also by the effective mass. The BT09 sample has a maximum PF of 6.66 mW mK^−2^ due to its high Seebeck coefficient and low resistivity (Figure 6e). This finding is also consistent with the investigation of optimal measurements of room temperature thermoelectric transport properties of n‐type Bi₂Te₃ by Witting et al.^[^ ^41^ ^]^ This is due to the high‐temperature treatment and Ti doping, which modulate the carrier concentration (nearly 10^20^ 1/cm^3^),^[^ ^40b^ ^]^ and keep the PF in the optimal range. The optimal combination for determining the PF is A3B3C2, as shown in Figure S10i,j (Supporting Information), which is consistent with the control parameter level for BT09. Therefore, BT09 is identified as the optimal sample under the best combination of control parameters. The PF value of 6.66 mW mK^−2^ is the highest reported so far for n‐type Bi_2_Te_3_‐based thin films prepared by magnetron sputtering. It is noteworthy that the PF values of all nine samples in this work are stable, reproducible, and higher than the previously reported PF values of sputtered thin films (Table S4, Supporting Information).^[^ ^30^ ^]^

## 3 Conclusion

In summary, we have prepared highly (015) optimally oriented nanocrystalline n‐type Bi_2_Te_3_ thin films with an average grain size of ≈110 nm and a high room‐temperature PF using high‐temperature sputtering and post‐annealing. Herein, the high‐temperature process generates Te vacancies, which create an electron‐like effect and increase the carrier concentration. This reduces carrier mobility by increasing the probability of lattice collisions, resulting in a lower velocity per unit number of electrons. Annealing improves the crystallinity of the film, increases the grain size, and reduces defects, thereby significantly increasing the mobility. In addition, Ti additives are pre‐deposited on the bottom of the film as an adhesion layer. At high temperatures, Ti ions are ionized and partially diffuse into Bi_2_Te_3_, inducing substitution defects between Ti and Bi, resulting in an electron‐effect that further increases the carrier concentration. It also enables a unique epitaxial growth of Bi_2_Te_3_ on the “transition layer” of TiTe_2_, resulting in highly (015) oriented films with enhanced electrical properties. By modulating the vacancy defects and introducing Ti additives, both the carrier concentration and the carrier mobility of the thin films can be increased simultaneously. As a result, the resistivity of the nine groups of n‐type Bi_2_Te_3_ thin films at room temperature ranges from 3.56 × 10^−6^ to 5.84 × 10^−6^ Ω∙m. The absolute value of the Seebeck coefficient ranges from 127.2 to 187.3 µV K^−1^, and the PF ranges from 4.54 to 6.66 mW mK^−2^. An n‐type Bi_2_Te_3_ thin film with a thickness of 5 µm achieves a PF of 6.66 mW mK^−2^ at room temperature, which is the highest value reported to date for an n‐type Bi_2_Te_3_ thin film by magnetron sputtering. Our work highlights the important effects of film thickness, annealing conditions and Ti additives on microstructure and charge carrier transport. More importantly, this work demonstrates the potential for large‐scale production of high‐quality Bi_2_Te_3_‐based thin films and devices for room temperature TE applications.

## 4 Experimental Section

### Sample Preparation

High‐resistance silicon <100> wafers with a diameter of 4 inches and a thickness of 400 µm were used as the substrate. Prior to use, the wafers were ultrasonically cleaned for 10 min in a beaker containing appropriate amounts of acetone, anhydrous ethanol, and deionized water. After cleaning, the wafers were dried with high purity nitrogen and stored in a clean container. The sputtering process was performed at a chamber pressure of 6 × 10^−3^ mbar. Magnetron sputtering was performed using a 99.999% pure n‐type Bi_2_Te_3_ alloy target and a 99.99% pure Ti target. First, a 50 nm thick Ti layer was deposited on the substrate surface at room temperature to serve as an adhesion material. The Bi_2_Te_3_ alloy was then sputtered at a substrate temperature of 270 °C.^[^ ^42^ ^]^ Sputtering was performed in RF mode with a power of 200 W and a sputtering rate of 4.95 Å s^−1^. Thin films with different thicknesses (1, 3, and 5 µm) were prepared by controlling the sputtering time. The samples were then subjected to a post‐annealing treatment in a rapid annealing furnace to reduce internal stresses and improve the crystalline properties of the thin films. The annealing process included different temperatures (250, 300, and 350 °C) and durations (1, 2, and 4 h).

### Characterization

The surface and cross‐sectional morphology of Bi_2_Te_3_ thin films were analyzed using a field emission scanning electron microscope (SEM‐Zeiss Sigma 300, Germany) with accelerating voltages ranging from 5 to 10 kV and amplitudes from 5 KX to 60 KX. The atomic compositions and ratios of the thin films were evaluated using an energy‐dispersive X‐ray spectroscopy (EDS) detector (Oxford Instruments, AztecLiveLite Xplore30, UK). The crystallographic properties of the composite films were determined using powder X‐ray diffraction (XRD) (Rigaku SmartLab, Japan) with Co‐Ka radiation (λ = 1.54056Å) at 45 kV and 100 mA. The 2theta‐omega diffraction angles ranged from 5° to 70° at a scanning speed of 0.02° per second. Tof‐SIMS (SIMS 5, Germany) analysis was performed using O_2_ sputtering elements with an energy of 2 keV and a positive ion collector with an area of 300 × 300 µm2. Transmission electron microscopy (TEM) was utilized for cross‐sectional HAADF‐STEM imaging and compositional profiling using a Thermo Scientific Helios 5 UC DualBeam instrument. Room‐temperature Hall measurements were performed to determine carrier concentration, carrier mobility, and electrical resistivity using a four‐point Van‐der‐Pauw arrangement (Lakeshore, 8400 Series HMS, USA). The Seebeck coefficients were measured using a physical property measurement system (Quantum Design, PPMS‐9, USA). Each measurement was averaged over 20 replicates.

### Optimization Method

Taguchi Design of Experiments was implemented using the software Minitab 19. Nine experimental schemes were designed based on the L9 array with the following three parameters and three levels selected: film thickness (1, 3, and 5 µm), annealing temperature (250, 300, and 350 °C), and annealing time (1, 2, and 4 h) (Tables S1 and S2, Supporting Information). The response parameters were carrier mobility, carrier concentration, resistivity, Seebeck coefficient, and power factor, which were determined experimentally. The sensitivity of the control parameters to the response parameters was assessed using the signal‐to‐noise (S/N) ratio. For carrier mobility, carrier concentration, Seebeck coefficient, and PF, the “larger is better” criterion is used, which can be expressed as follows

(3) $$Larger\;is\;better\;S/N\left(dB\right)=-10\log_{10}\left[\frac{1}{R}\sum_{i=1}^{R}\frac{1}{y_{i}^{2}}\right]$$

For electrical resistivity, it is necessary to reduce it as much as possible, so the “smaller is better” criterion is used, which can be calculated as follows

(4) $$Smaller\;is\;better\;S/N\left(dB\right)=-10\log_{10}\left[\frac{1}{R}\sum_{i=1}^{R}y_{i}^{2}\right]$$

where *R* is the number of repetitions for each trial and *y*_i_ is the output response value for the *i*_th_ trial. The difference between the maximum and minimum S/N ratios of the three factors at the three levels was used as a measure of their significance effect (Figure S10, Supporting Information). The maximum output response can be obtained by the optimal combination of control parameters.

### Density Functional Theory Calculation

The fully self‐consistent ab initio calculations for bulk compounds were carried out in the framework of Density Functional Theory (DFT). The pseudo‐potential of the local density approximation (LDA) was employed to calculate the electronic energy band structure. To ensure convergence, a cutoff energy of 340 eV was fixed for the wavefunction expansion and a 10 × 10 × 10 k‐point mesh was used in the BZ. Spin‐orbit coupling (SOC) was considered and the conjugate gradient (CG) algorithm was used to relax the lattice and all atoms.

## Conflict of Interest

The authors declare no conflict of interest.

## Supporting information

Supporting Information

## Data Availability

The data that support the findings of this study are available from the corresponding author upon reasonable request.
