# Supplementary material for: Ultrahigh Power Factor of Sputtered Nanocrystalline N‐Type Bi2Te3 Thin Film via Vacancy Defect Modulation and Ti Additives
Source: Adv Sci (Weinh). 2024 Aug 9;11(38):2403845. doi: 10.1002/advs.202403845 (PMC11481191; doi:10.1002/advs.202403845)
Supplement: Supplementary file 1 — Supporting Information [file ADVS-11-2403845-s001.docx]

Supporting Information

Ultrahigh Power Factor of Sputtered Nanocrystalline N-type Bi_2_Te_3_ Thin Film via Vacancy Defect Modulation and Ti Additives

Tingrui Gong*, Lei Gao, Lingfeng Kang, Maolin Shi, Gu Hou, Shenghui Zhang, Dechao Meng, Juntao Li, Wei Su*

T. Gong, L. Gao, L. Kang, M. Shi, G. Hou, S. Zhang, D. Meng, J. Li

Microsystem & Terahertz Research Center

China Academy of Engineering Physics

Chengdu, Sichuan 610200, China
E-mail: gongtingrui_mtrc@caep.cn

T. Gong, L. Gao, L. Kang, M. Shi, G. Hou, S. Zhang, D. Meng, J. Li, W. Su
Institute of Electronic Engineering

China Academy of Engineering Physics

Mianyang, Sichuan 621999, China

E-mail: weisu@caep.cn


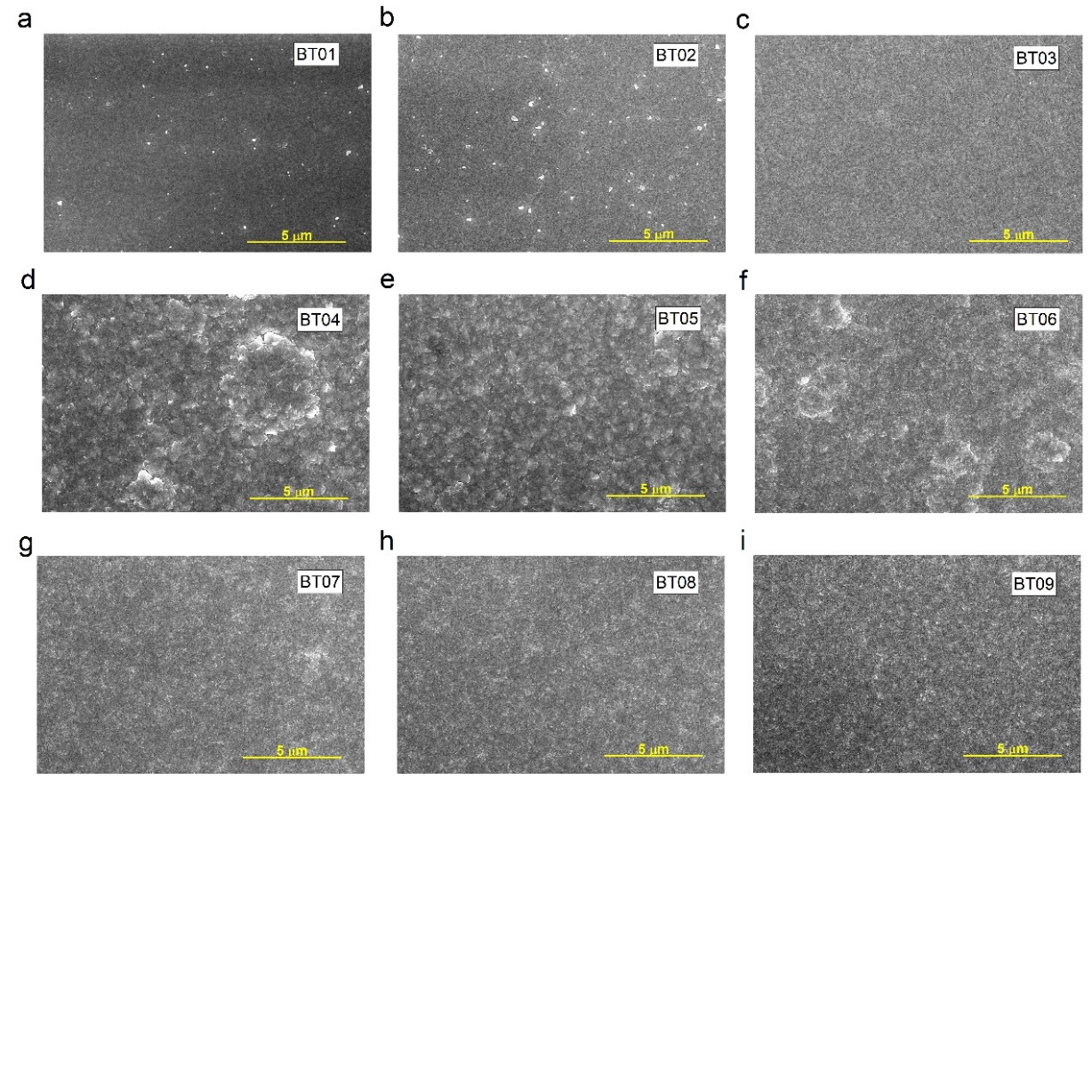


**Figure S1.** Surface SEM images of the nine Bi_2_Te_3_ thin films at a scale of 5 µm: (a) BT01. (b) BT02. (c) BT03. (d) BT04. (e) BT05. (f) BT06. (g) BT07. (h) BT08. (i) BT09.


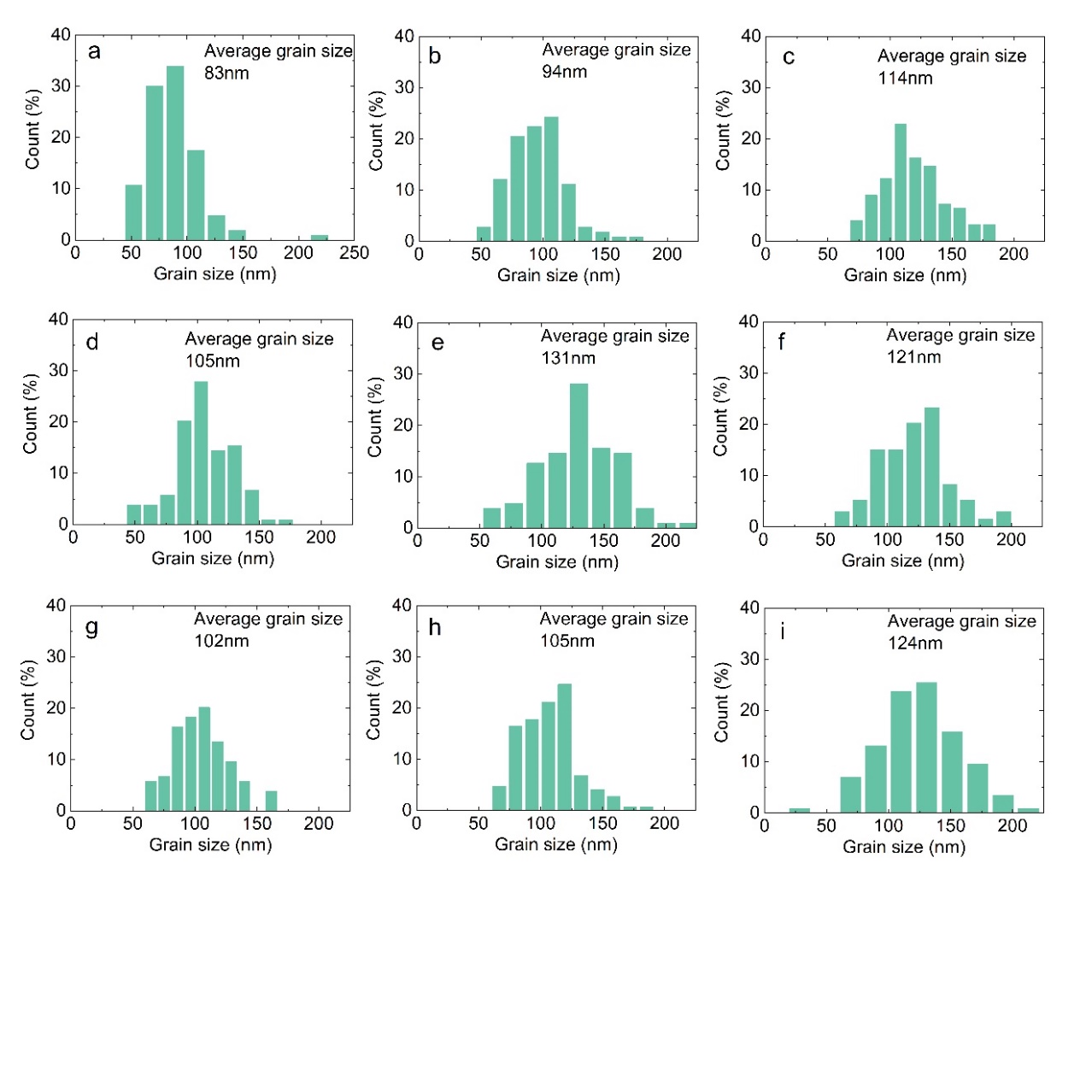


**Figure S2.** Grain size distributions of the nine Bi_2_Te_3_ thin films: (a) BT01. (b) BT02. (c) BT03. (d) BT04. (e) BT05. (f) BT06. (g) BT07. (h) BT08. (i) BT09.

**Figure S3.** Grain size comparisons of the nine Bi_2_Te_3_ thin films.


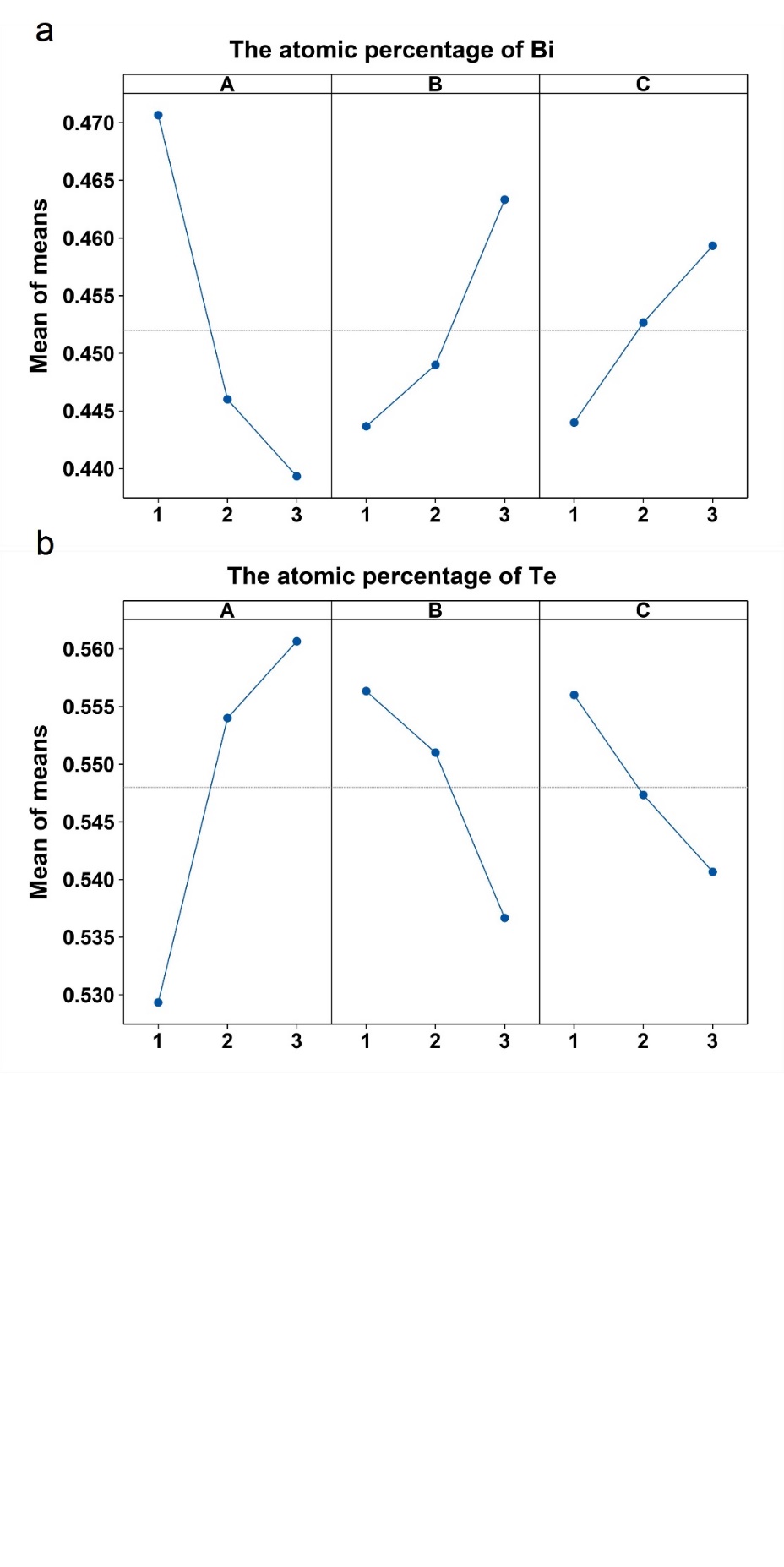


**Figure S4.** The mean main effects of the control parameters on the thin film compositions.

**Figure S5.** Tof-SIMS spectrum.


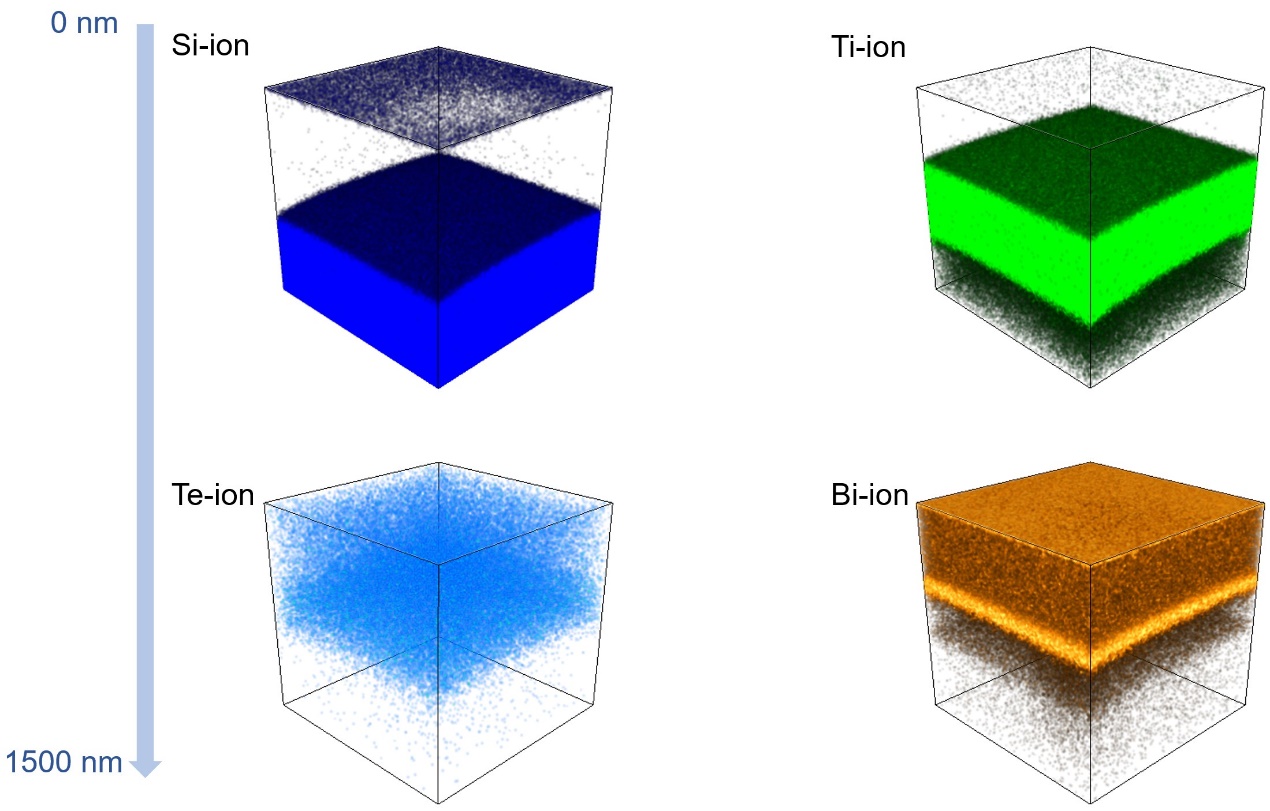


**Figure S6.** Tof-SIMS mapping.


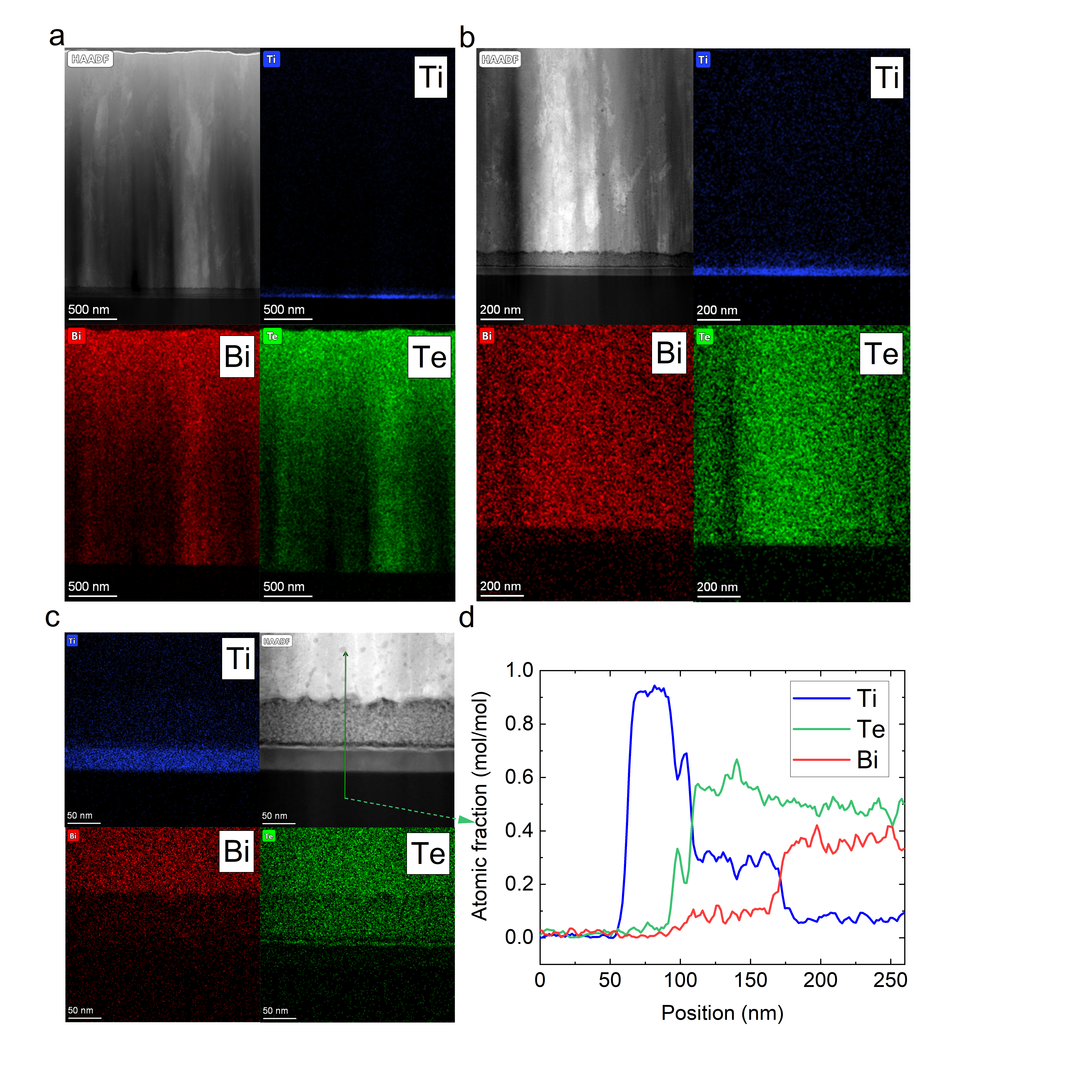


**Figure S7.** Cross-sectional HAADF-STEM images of the BT09 sample. (a) STEM HAADF images and corresponding EDS mapping at a scale of 500 nm. (b) STEM HAADF images and corresponding EDS mapping at a scale of 200 nm. (c) STEM HAADF images and corresponding EDS mapping at a scale of 50 nm, showing that the diffused region is rich in Te but almost devoid of Bi. (d) Compositional profile along the arrow line, showing that the atomic ratio of Te to Ti is close to 2:1.

**Figure S8.** The XRD patterns of BT03 and BT09 are taken in logarithmic longitudinal coordinates. The film thickness of the BT03 sample is 1 µm, which makes TiTe_2_ easier to detect than the 5 µm thickness of the BT09 sample.


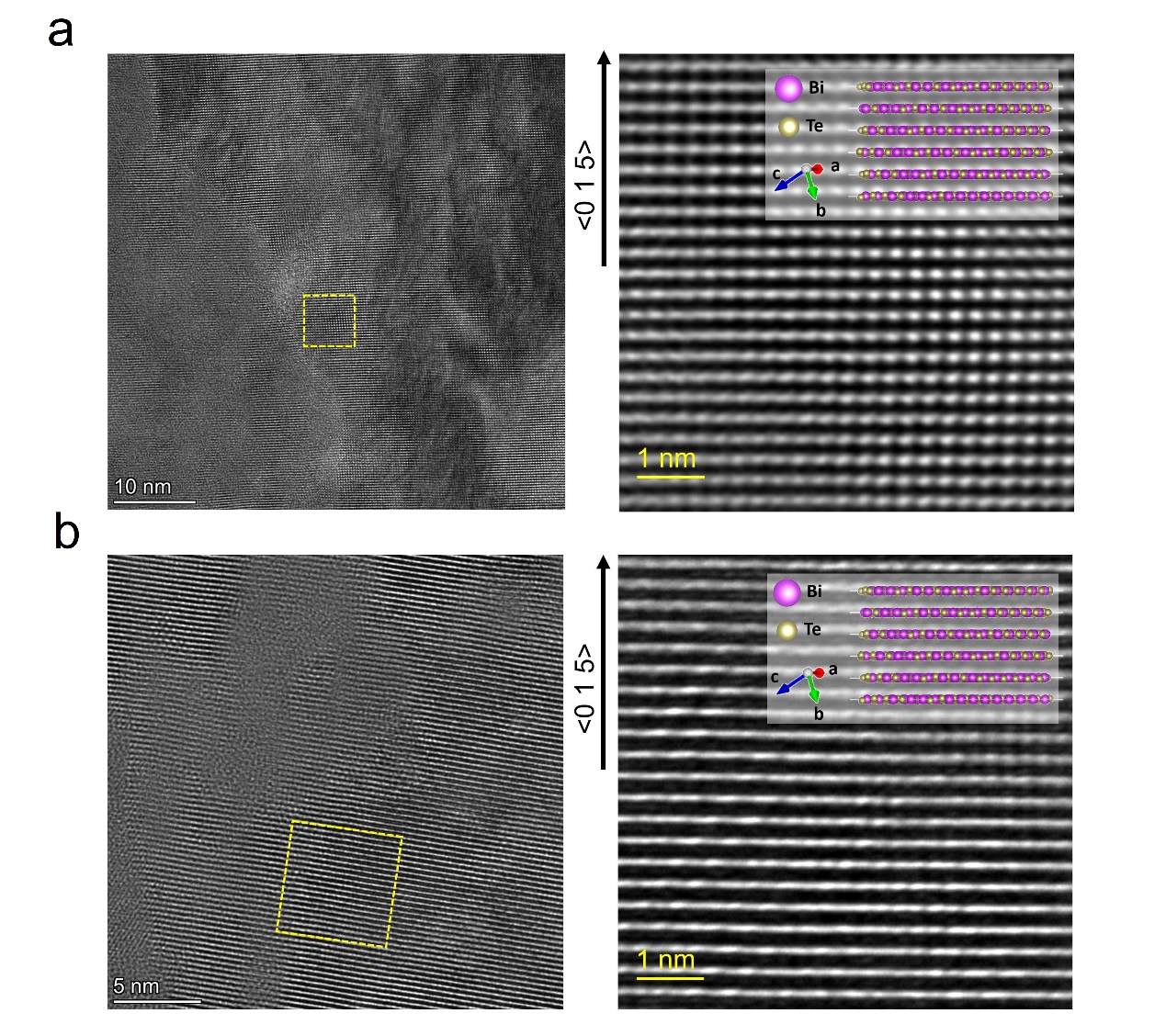


**Figure S9.** (a) High Resolution TEM (HRTEM) image of the BT03 sample. (b) The enlarged image of marked region in (a). (c) High Resolution TEM (HRTEM) image of the sample BT09. (d) The enlarged image of marked region in (c).





**Figure S10.** Mean of S/N ratios and factor effects of the nine Bi_2_Te_3_ thin films with different responses. (a, b) Carrier mobility. (c, d) Carrier concentration. (e, f) Resistivity. (g, h) Seebeck coefficient. (i, j) Power factor.

**Figure S11.** Comparison of electrical transport properties of Bi_2_Te_3_ thin films with and without Ti additives. These samples were sputtered simultaneously, all with a thickness of 1 µm. The annealing conditions were constant, with an annealing temperature of 350 °C and an annealing time of 4 hours. (a) Carrier mobility. (b) Carrier concentration. (c) Resistivity.

**Table S1.** Selected parameters and levels for Taguchi method optimization.

| Label | Parameter | Level |  |  |
| --- | --- | --- | --- | --- |
|  |  | 1 | 2 | 3 |
| A | Film thickness (μm) | 1 | 3 | 5 |
| B | Annealing temperature (ºC) | 250 | 300 | 350 |
| C | Annealing time (hour) | 1 | 2 | 4 |

**Table S2.** Orthogonal array based on Taguchi method.

| Scheme | Film thickness (μm) | Annealing temperature (ºC) | Annealing time (hour) |
| --- | --- | --- | --- |
| BT01 | 1 | 250 | 1 |
| BT02 | 1 | 300 | 2 |
| BT03 | 1 | 350 | 4 |
| BT04 | 3 | 250 | 2 |
| BT05 | 3 | 300 | 4 |
| BT06 | 3 | 350 | 1 |
| BT07 | 5 | 250 | 4 |
| BT08 | 5 | 300 | 1 |
| BT09 | 5 | 350 | 2 |

**Table S3.** Atomic compositions and ratios of the nine Bi_2_Te_3_ thin films.

| Scheme | Bi (%) | Te (%) |
| --- | --- | --- |
| BT01 | 45.0 | 55.0 |
| BT02 | 46.3 | 53.7 |
| BT03 | 49.9 | 50.1 |
| BT04 | 44.8 | 55.2 |
| BT05 | 44.6 | 55.4 |
| BT06 | 44.4 | 55.6 |
| BT07 | 43.3 | 56.7 |
| BT08 | 43.8 | 56.2 |
| BT09 | 44.7 | 55.3 |

**Table S4.** Lattice constant and band gap variation of Ti-doped Bi_2_Te_3_

|  | a (Å) | c (Å) | Angle $\boldsymbol{\alpha}$ (degree) | Angle $\boldsymbol{\gamma}$ (degree) | E_g_ (eV) |
| --- | --- | --- | --- | --- | --- |
| Bi_2_Te_3_ | 4.386 | 30.497 | 90 | 120 | 0.095 |
| BiTiTe_3_ | 4.453 | 30.702 | 90 | 120 | 0.221 |

**Table S5.** Summary of N-type Bi_2_Te_3_ thin film fabricated by magnetron sputtering

| TE material | Thickness (μm) | Resistivity (Ω∙cm) | Seebeck coefficient (μV/K) | Power factor (mW/mK^2^) | Refs. |
| --- | --- | --- | --- | --- | --- |
| Bi_2_Te_3_ | 1 | 1.19×10^-3^ | -200 | 3.37 | [[1](#_ENREF_1)] |
| Bi_2_Te_3_ | 0.4 | 2.78×10^-3^ | -242 | 2.1 | [[2](#_ENREF_2)] |
| Bi_2_Te_3_ | 0.3 | 3.85×10^-4^ | -70 | 1.27 | [[3](#_ENREF_3)] |
| Bi_2_Te_3_ | 1 | 9.76×10^-4^ | -163 | 2.73 | [[4](#_ENREF_4)] |
| Bi_2_Te_3_ | 0.8 | 1.18×10^-3^ | -76 | 0.49 | [[5](#_ENREF_5)] |
| Bi_2_Te_3_ | 0.6 | 3.23×10^-3^ | -162.4 | 0.817 | [[6](#_ENREF_6)] |
| Bi_2_Te_3_ | 8 | 1.56×10^-3^ | -102 | 0.7 | [[7](#_ENREF_7)] |
| Bi_2_Te_3_ | 1 | 1.82×10^-3^ | -128 | 0.9 | [[8](#_ENREF_8)] |
| Bi_2_Te_3_ | 10 | 1.82×10^-3^ | -123 | 0.83 | [[9](#_ENREF_9)] |
| Bi_2_Te_3_ | 0.6 | 8.93×10^-4^ | -195 | 4.25 | [[10](#_ENREF_10)] |
| Bi_2_Te_3_ | 1 | 1.65×10^-3^ | -162.99 | 1.61 | [[11](#_ENREF_11)] |
| Bi_2_Te_3_ | 1.45 | 2.8×10^-4^ | -87.7 | 2.75 | [[12](#_ENREF_12)] |
| BT01 | 1 | 3.73×10^-4^ | -139.6 | 5.22 | This work |
| BT02 | 1 | 3.56×10^-4^ | -127.2 | 4.54 | This work |
| BT03 | 1 | 3.83×10^-4^ | -145.2 | 5.50 | This work |
| BT04 | 3 | 4.76×10^-4^ | -164.4 | 5.68 | This work |
| BT05 | 3 | 4.44×10^-4^ | -159.3 | 5.72 | This work |
| BT06 | 3 | 4.45×10^-4^ | -152.9 | 5.25 | This work |
| BT07 | 5 | 5.84×10^-4^ | -177.5 | 5.39 | This work |
| BT08 | 5 | 5.38×10^-4^ | -182.7 | 6.20 | This work |
| BT09 | 5 | 5.27×10^-4^ | -187.3 | 6.66 | This work |

Reference

[1] Y. Deng, Z. Zhang, Y. Wang, Y. Xu, *Journal of Nanoparticle Research* **2012**, *14*, 1.

[2] X. Wang, H. He, N. Wang, L. Miao, *Applied Surface Science* **2013**, *276*, 539.

[3] Y. Zhou, L. Li, Q. Tan, J.-F. Li, *Journal of Alloys and Compounds* **2014**, *590*, 362.

[4] T. Kurokawa, R. Mori, O. Norimasa, T. Chiba, R. Eguchi, M. Takashiri, *Vacuum* **2020**, *179*, 109535.

[5] Z. He, Y.-X. Chen, Z. Zheng, F. Li, G. Liang, J. Luo, P. Fan, *Ceramics International* **2020**, *46* (9), 13365.

[6] J. Zhang, X. Jia, H. Yan, N. Xiao, P. Ning, L. Yu, Y. Li, *AIP Advances* **2023**, *13* (2).

[7] M. T. Dunham, M. T. Barako, J. E. Cornett, Y. Gao, S. Haidar, N. Sun, M. Asheghi, B. Chen, K. E. Goodson, *Advanced Materials Technologies* **2018**, *3* (6), 1700383.

[8] D.-H. Kim, G.-H. Lee, *Materials Science and Engineering: B* **2006**, *131* (1-3), 106.

[9] S. A. Haidar, Y. Gao, Y. He, J. E. Cornett, B. Chen, N. J. Coburn, C. Glynn, M. T. Dunham, K. E. Goodson, N. Sun, *Thin Solid Films* **2021**, *717*, 138444.

[10] C. Yun-Fei, W. Feng, W. He, Z. Wei-Yun, D. Yuan, *ACTA PHYSICA SINICA* **2021**, *70* (20).

[11] O. Norimasa, T. Chiba, M. Hase, T. Komori, M. Takashiri, *Journal of Alloys and Compounds* **2022**, *898*, 162889.

[12] S. Kianwimol, P. Wanarattikan, R. Sakdanuphab, P. Pluengphon, T. Bovornratanaraks, A. Sakulkalavek, *Journal of Electronic Materials* **2019**, *48*, 3490.
